# Further Characterisation of the Translational Termination-Reinitiation Signal of the Influenza B Virus Segment 7 RNA

**DOI:** 10.1371/journal.pone.0016822

**Published:** 2011-02-08

**Authors:** Michael L. Powell, Kendra E. Leigh, Tuija A. A. Pöyry, Richard J. Jackson, T. David K. Brown, Ian Brierley

**Affiliations:** 1 Division of Virology, Department of Pathology, University of Cambridge, Cambridge, United Kingdom; 2 Department of Biochemistry, University of Cambridge, Cambridge, United Kingdom; Victor Chang Cardiac Research Institute (VCCRI), Australia

## Abstract

Termination-dependent reinitiation is used to co-ordinately regulate expression of the M1 and BM2 open-reading frames (ORFs) of the dicistronic influenza B segment 7 RNA. The start codon of the BM2 ORF overlaps the stop codon of the M1 ORF in the pentanucleotide **UAA** UG and ∼10% of ribosomes terminating at the M1 stop codon reinitiate translation at the overlapping AUG. BM2 synthesis requires the presence of, and translation through, 45 nt of RNA immediately upstream of the **UAA** UG, known as the ‘termination upstream ribosome binding site’ (TURBS). This region may tether ribosomal 40S subunits to the mRNA following termination and a short region of the TURBS, motif 1, with complementarity to helix 26 of 18S rRNA has been implicated in this process. Here, we provide further evidence for a direct interaction between mRNA and rRNA using antisense oligonucleotide targeting and functional analysis in yeast cells. The TURBS also binds initiation factor eIF3 and we show here that this protein stimulates reinitiation from both wild-type and defective TURBS when added exogenously, perhaps by stabilising ribosome-mRNA interactions. Further, we show that the position of the TURBS with respect to the **UAA** UG overlap is crucial, and that termination too far downstream of the 18S complementary sequence inhibits the process, probably due to reduced 40S tethering. However, in reporter mRNAs where the restart codon alone is moved downstream, termination-reinitiation is inhibited but not abolished, thus the site of reinitiation is somewhat flexible. Reinitiation on distant AUGs is not inhibited in eIF4G-depleted RRL, suggesting that the tethered 40S subunit can move some distance without a requirement for linear scanning.

## Introduction

Eukaryotic viruses have evolved a variety of translational control strategies to facilitate expression of downstream open reading frames (ORFs) on polycistronic mRNAs and examples have been described at all three steps of protein synthesis - initiation, elongation and termination [1]. These include leaky scanning of 40S ribosomal subunits past the most 5′ AUG [2], the *de novo* recruitment of ribosomes to intercistronic internal ribosomal entry sites (IRESs; [3]); programmed ribosomal frameshifting [4], [5] and the circumvention of normal termination by programmed stop codon readthrough [6], [7]. Generally, these processes allow the expression of two (or more) proteins from a single mRNA and may also permit a level of control over their relative quantities. Another way of accessing a downstream ORF in viral mRNAs is by termination-dependent reinitiation of translation (termination-reinitiation), a phenomenon first described in the expression of the influenza B virus BM2 protein [8]. The dicistronic mRNA that is derived from genomic segment 7 of this virus has two ORFs encoding matrix protein 1 (M1) and BM2, with the termination codon of M1 in close proximity to the start codon of the BM2 ORF (**UAA** UG; stop codon of M1 in bold, start codon of BM2 underlined) [8]–[10]. Following translation of M1, some 10–20% of ribosomes terminating at the M1 stop codon go on to reinitiate translation at the immediately adjacent BM2 start codon [8], [11]. This capacity to reinitiate protein synthesis following translation of a long upstream ORF was unexpected. During the elongation phase, initiation factors are likely to be rapidly lost, thus reinitiation of translation following termination was believed to be restricted to cases where the upstream ORF (uORF) is very short [12]–[14]. Our knowledge of the mRNA signals that allow efficient reinitiation following translation of a long upstream ORF has largely been obtained from studies of caliciviruses, namely in the expression of the VP2 protein of feline calicivirus (FCV; [15]–[17]) and the VP10 protein of rabbit haemorrhagic disease virus (RHDV; [18], [19]). Here, expression of the downstream ORF by termination-reinitiation requires a stretch of mRNA (between 69 and 87 nt in length) upstream of the stop codon of the first ORF (termed the termination upstream ribosome binding site or TURBS) and the close proximity of the stop and start codons of the two ORFs [15], [17], [19]. Within the TURBS, two essential sequence motifs (motifs 1 and 2) have been described. Motif 1 is highly conserved amongst caliciviruses and is composed of a short stretch of 6–9 nt (with a conserved core of UGGGA) with complementarity to the apical loop of helix 26 of 18S rRNA. This motif, which lies towards the 5′ boundary of the TURBS, likely acts to tether the 40S subunit to the mRNA post-termination, facilitating the recruitment of the initiation factors necessary for reinitiation [15], [19]. Motif 2, which lies some 15–20 nt upstream of the termination-reinitiation window (A**UG** **A** in FCV), is a second essential stretch of 4–8 nt but shows little sequence conservation amongst caliciviruses. Recent work has revealed that this motif in fact forms the 3′ arm of a stem-loop structure whose formation is necessary for efficient termination-reinitiation [16].

The features identified in caliciviral TURBS suggest a model for termination-reinitiation in which post-termination 40S subunits are tethered to the mRNA through interactions between the mRNA (through motif 1) and 18S rRNA, initiation factors are recruited and the AUG restart codon located, processes which may require precise RNA folding (involving motif 2) within the TURBS. The TURBS is not highly active as an IRES and 40S subunit recruitment probably requires high local 40S subunit concentrations [17], [18]. An important question, therefore, is how the interaction between TURBS motif 1 and 18S rRNA helix 26 is facilitated. Analysis of primary and secondary structural features of the BM2 signal has revealed that it contains a short TURBS (of 45 nt) which is largely single-stranded, with motif 1 likely to be located in the apical loop of a metastable stem-loop structure when the ribosome is positioned at the termination codon of the upstream ORF [11]. We have suggested that motif 1 may thus be “presented” to the solvent-accessible apical loop of helix 26, promoting the interaction and subsequent 40S tethering. However, this hypothesis remains contentious. The paucity of stable RNA secondary structure within the BM2 TURBS [11], the TURBS of FCV [I. Brierley, unpublished observations] and the calicivirus murine norovirus (MNV; [20]), limits the predictive power of RNA folding programs in the assessment of the likely RNA folds present before and after ribosomal transit through the TURBS. It is also known that eukaryotic initiation factor 3 (eIF3) plays a role in the termination-reinitiation process, perhaps interacting with the TURBS to provide another link between mRNA and the 40S subunit [17].

In this paper, we describe further analysis of the BM2 TURBS. In contrast to the caliciviral TURBS, which contain stretches of non-essential bases between motifs 1 and 2, all of the BM2 TURBS appears to be required for function perhaps due to its shorter length, with sequence-specific and sequence-independent elements. Evidence is provided, from oligonucleotide targeting and from expression studies in yeast cells, to support the hypothesis that BM2 motif 1 interacts directly with helix 26 of 18S rRNA, consistent with a tethering role. We also provide evidence that a close spacing between the M1 stop and BM2 start codons is not critical; rather, there is dependence upon the distance between the terminating ribosome and the TURBS. Indeed, the ribosome is able to locate start codons placed some distance downstream of the wild-type position if termination occurs at the normal distance relative to the TURBS. The role of RNA secondary structure in TURBS function was also investigated by mutagenesis, but the experiments gave only limited support for the predicted secondary structures. However, the capacity of eIF3 to stimulate termination-reinitiation, particularly from a defective TURBS, was confirmed, including a TURBS rendered defective through a point mutation in motif 1. Together, these data support the view that efficient termination-reinitiation requires both mRNA-rRNA interactions and the participation of eIF3.

## Materials and Methods

### Construction of plasmids

The p2luc-BM2 plasmid series was prepared by subcloning sequences encompassing the influenza B virus termination-reinitiation signal (prepared by RT-PCR) into the dual-luciferase reporter plasmid p2luc [21]. Most of these plasmids have been described previously [11]. The p2luc-BM218S-AG, 18S-AT and 18S-AC plasmids were generated by site directed mutagenesis of p2luc-BM2wt or p2luc-BM2-204 (see below).

The CrPV-p2luc-BM2 plasmid series was generated by digestion of p2luc-BM2wt with *PstI* and *NheI*, and insertion of a PCR fragment comprising the cricket paralysis virus (CrPV) IRES downstream of a bacteriophage T7 promoter. The T7-CrPV fragment was generated by PCR from plasmid CrPV/+8norm [14]. The resulting plasmid contains a T7 promoter located 25 nt upstream of the CrPV IRES. Translation starts on an alanine codon within the inserted CrPV fragment, resulting in an N-terminal extension to *rluc* of 10 amino acids relative to that of the p2luc-BM2wt. Derivative plasmids were generated by site-directed mutagenesis using primers described previously [11].

Termination-reinitiation in yeast cells was studied using the yeast dual-reporter plasmid pAC99 [22], [23]. The wild-type termination-reinitiation signal of BM2 was prepared by PCR as an *EcoRV* fragment that was subsequently cloned into *MscI*-digested pAC99. pAC99-BM2 derivative plasmids were generated by site directed mutagenesis as described below.

Sequences were confirmed by commercial dideoxy sequencing (using the facility at the Department of Biochemistry, University of Cambridge).

### Site-directed mutagenesis

Site-directed mutagenesis was performed using the Quikchange II site-directed mutagenesis kit (Stratagene) according to manufacturer's instructions. Mutagenesis to introduce insertions longer than 6 bp was performed in two steps [24], by first subjecting the mutagenesis reactions (containing either the sense or antisense primer) to three cycles of PCR, then mixing the reactions and performing a further 18 cycles as previously described [11].

### *In vitro* transcription and translation

p2luc-BM2 reporter plasmids were linearised with *HpaI* and capped run-off transcripts generated using T7 RNA polymerase as described previously [25]. Messenger RNAs were recovered by a single extraction with phenol/chloroform (1∶1 *v/v*) followed by ethanol precipitation. Remaining unincorporated nucleotides were removed by gel filtration through a NucAway spin column (Ambion). The eluate was concentrated by ethanol precipitation, the mRNA resuspended in water, checked for integrity by agarose gel electrophoresis and quantified by spectrophotometry.

Unless otherwise stated, mRNAs were translated in Flexi® rabbit reticulocyte lysate (Flexi®RRL, Promega) programmed with template mRNA at 50 µg/ml. Typical reactions were of 10 µl and composed of 60% (v/v) Flexi®RRL, 20 µM amino acids (lacking methionine), 500 µM MgOAc, 2 mM DTT, 5U RNase inhibitor (RNAguard, GE Healthcare Life Sciences), 130 mM–160 mM KCl (optimised for each batch of Flexi®RRL) and 0.2 MBq [^35^S]-methionine. Reactions were incubated for 1 h at 30°C and stopped by the addition of an equal volume of 10 mM EDTA, 100 µg/ml RNase A followed by incubation at room temperature for 20 minutes. Samples were prepared for SDS-PAGE by the addition of 4 volumes of 4X Laemmli's sample buffer, boiled for 3 minutes and resolved on 12% SDS-PAGE gels. The relative abundance of products on the gels was determined by direct measurement of [^35^S]-methionine incorporation using a Packard Instant Imager 2024. eIF4G-depleted RRL was prepared and used as described previously [26].

### Proteins and cell extracts

Purified eIF3 was a kind gift of Dr. Chris Fraser (Department of Molecular and Cell Biology and Howard Hughes Medical Institute, University of California). Dominant-negative eIF4A-R362Q was prepared as described [14].

### Reporter gene assay in yeast cells

pAC99 reporter plasmids were transformed into yeast strain Y349 using the LiOAc/ssDNA/PEG method [27]. For each experiment three transformants cultivated under the same conditions were assayed. Cells were disrupted by vortexing with acid washed glass beads (Sigma) at 4°C for 30 minutes. Cell debris was removed by centrifugation and reporter enzyme assays carried out as described [23]. Termination-reinitiation efficiency was calculated as the ratio of firefly luciferase activity to β-galactosidase activity relative to that of constructs in which the open reading frames were fused in frame.

## Results

### The BM2 TURBS is streamlined into 45nt of RNA essential for termination-reinitiation, containing sequence-specific and sequence-independent elements

**Figure 1:**
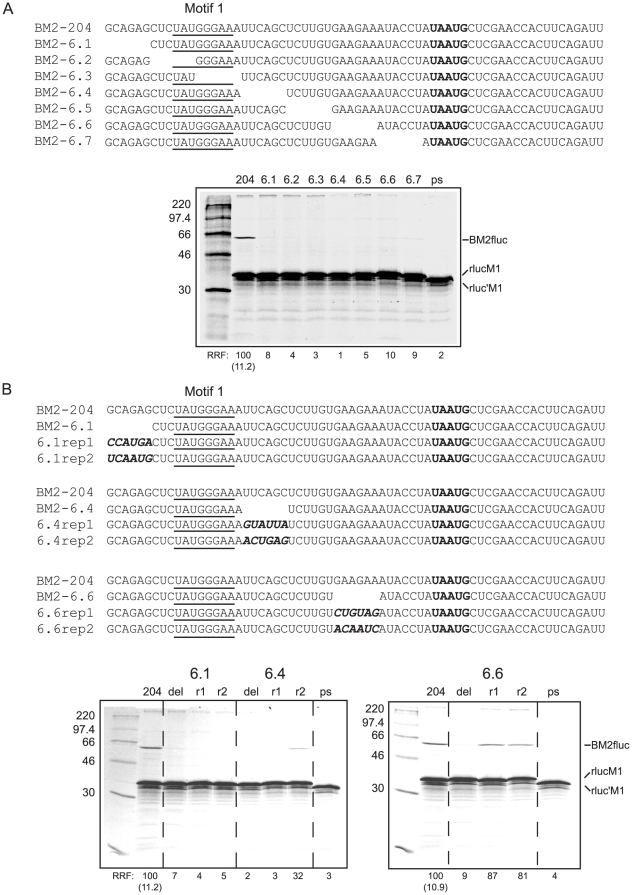
Deletion analysis of the BM2 TURBS. (A) The primary sequence of the TURBS of the wild-type reporter mRNA (BM2-204) and each of the 6 nt ‘moving window’ deletion mutants is shown. The **UAA** UG overlap is highlighted in bold and motif 1 is underlined in BM2-204. Plasmids were linearised with *HpaI*, transcribed and the mRNA translated in Flexi® RRL (in the presence of [^35^S]-methionine) supplemented with 140 mM KCl. The products were resolved by SDS-PAGE and visualised by autoradiography. The ∼36 kDa band (in all translations apart from ps where the introduced stop codon reduces its length to ∼33 kDa) corresponds to the upstream rlucM1 ORF, the ∼62 kDa band is the BM2fluc termination-reinitiation product. Translations of the deletion mutant mRNAs were performed in parallel with relevant controls (204, ps). (B) The TURBS of three selected 6 nt deletion mutants is shown relative to BM2-204, and the replacement mutants (rep1 or rep2) are shown underneath. Inserted nucleotides are shown in bold italics, and the termination-reinitiation overlap is shown in bold. Translations of the 6 nt deletion mutants (del) and the replacement mutants (r1 and r2) were performed in parallel with the 204 and ps controls, resolved on SDS-PAGE and visualised by autoradiography.

In RHDV and FCV, TURBS motifs 1 and 2 are separated by some 30 nt, much of which appears to be non-essential [15]–[17], [19]. However, given that the minimal sequence requirement for termination-reinitiation of BM2 (∼45 nt; [11]) is shorter than that documented in the caliciviruses (69 and 87 nt; [15], [17], [19]), we wished to test whether any of the residues were non-essential. To do this, we introduced 6 nt deletions throughout the length of the sequence upstream of the M1 termination codon in the context of the BM2 termination-reinitiation reporter plasmid, p2luc-BM2-204 (Figure 1A; [11]). As expected, translation of the BM2-204 reporter mRNA generated products corresponding to the cap-dependent upstream product (rlucM1-204 ∼36 kDa) and the termination-reinitiation product (BM2fluc ∼62 kDa). The latter protein was not observed in translations of the negative control reporter mRNA, p2luc-BM2ps (rluc'M1 ∼33 kDa, [11]), which contains an in-frame stop codon immediately upstream of the TURBS. Significantly, deletion of any part of the minimal required region led to a strong inhibition of BM2fluc synthesis, suggesting that the entire TURBS is required for efficient termination-reinitiation on the segment 7 RNA (Figure 1A). Alternatively, it was possible that the defect in BM2 synthesis in this deletion series may be a consequence of the altered spacing between the M1 stop codon and upstream features required for termination-reinitiation. To test this, we selected three of the 6 nt deletion mutants surrounding motif 1 (mutants 6.1, 6.4 and 6.6) and replaced the deleted regions with two different random 6 nt sequences (Figure 1B). No restoration of BM2 synthesis was observed with either of the 6.1 nucleotide replacement mutants (Figure 1B). Replacement of nucleotides into the 6.4 deletion mutant had no effect with the first of the mutants (6.4r1), but some recovery was observed in the second of these mutants (6.4r2), albeit the frequency of termination-reinitiation was diminished compared to that of the wild-type mRNA (Figure 1B). In contrast, insertion of 6 nt back into the 6.6 deletion mutant fully restored BM2 synthesis in both cases (Figure 1B). These results suggest that the minimal 45 nt TURBS region is split into both sequence-specific and sequence-independent elements, where the sequence towards the 5′ end of the minimal region is important in base-specific interactions, whereas the 3′ end of the TURBS may act solely as a spacer, important in placing motif 1 relative to the terminating ribosome.

### Targeting the 18S rRNA:mRNA interaction using antisense oligonucleotides

**Figure 2:**
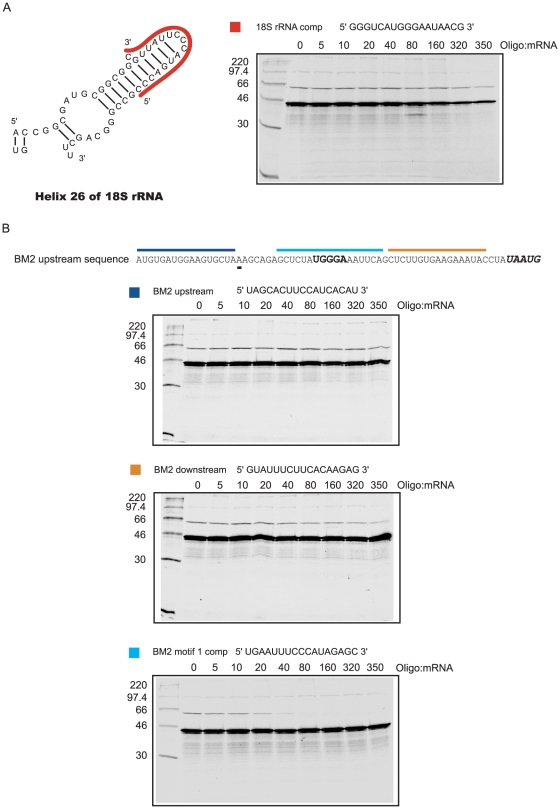
An antisense RNA oligonucleotide specifically inhibits the BM2 termination-reinitiation process. (A) The secondary structure of helix 26 of murine 18S rRNA is shown with the AON targeted region (which has the same sequence in rabbit) illustrated with a red line. The primary sequence of the oligonucleotide is also shown. The BM2-wt RNA was translated in Flexi® RRL in the presence of increasing concentrations of the AON. The 44 kDa product produced from this mRNA corresponds to the rlucM1 product, the 62 kDa product is the BM2fluc reinitiation product. The molar excess of AON (oligo) in relation to the mRNA is shown above the autoradiograph. (B) The sequence upstream of the termination-reinitiation site (**UAA** UG) is shown, with the A residue at the start of the TURBS underlined. Also shown is the core sequence of motif 1 in bold. The regions targeted by AONs are shown above the sequence, colour coded to match the relevant AON sequence. The BM2wt RNA was translated as above in the presence of increasing AON concentrations as indicated above each autoradiograph.

Termination-reinitiation on the BM2 ORF is likely to be dependent on interactions between the influenza B segment 7 RNA and helix 26 of the 18S rRNA [11]. However, other work has revealed that motif 1 may also be important in eIF3 binding [17]. To investigate the mRNA-18S rRNA interaction further, antisense 2-*O*-methyl oligonucleotides (AONs) were synthesised that would target either the loop of helix 26 of 18S rRNA (Figure 2A) or motif 1 of the BM2 TURBS (Figure 2B). Control oligonucleotides were also prepared to target sequences both upstream and downstream of motif 1 to control for potential non-specific effects on translation (Figure 2B). An AON designed to target helix 26 of the 18S rRNA had no effect on BM2 synthesis (Figure 2A), although high concentrations (320-fold to 2960-fold molar excess of the AON to the mRNA) inhibited global translation (Figure 2A and data not shown). Conversely, an AON targeting motif 1 specifically inhibited BM2 synthesis, with little effect on overall translation (Figure 2B). Importantly, the upstream and downstream control AONs had little effect on BM2 synthesis or global translation within the range tested for the BM2 motif 1 complementary AON (Figure 2B). It should be noted that the 80S ribosome will strip annealed AONs from the mRNA as it translates through the TURBS. However, both the upstream and motif 1 complementary AONs will be able to re-anneal once the ribosome reaches the **UAA** UG. However, it is possible that any effect of the downstream oligo is masked due to it being unable to reanneal in the presence of the terminating ribosome. Nevertheless, these data indicate that the effect of the BM2 motif 1 complementary AON is specific and not an effect on ribosome processivity, and provide further evidence in support of an interaction between motif 1 and 18S rRNA. However, we cannot rule out that these effects may also be due to perturbations of RNA secondary structure within the TURBS or due to interactions of motif 1 with an unspecified molecule (see later).

### Investigation of 18S complementary elements involved in termination-reinitiation

**Figure 3:**
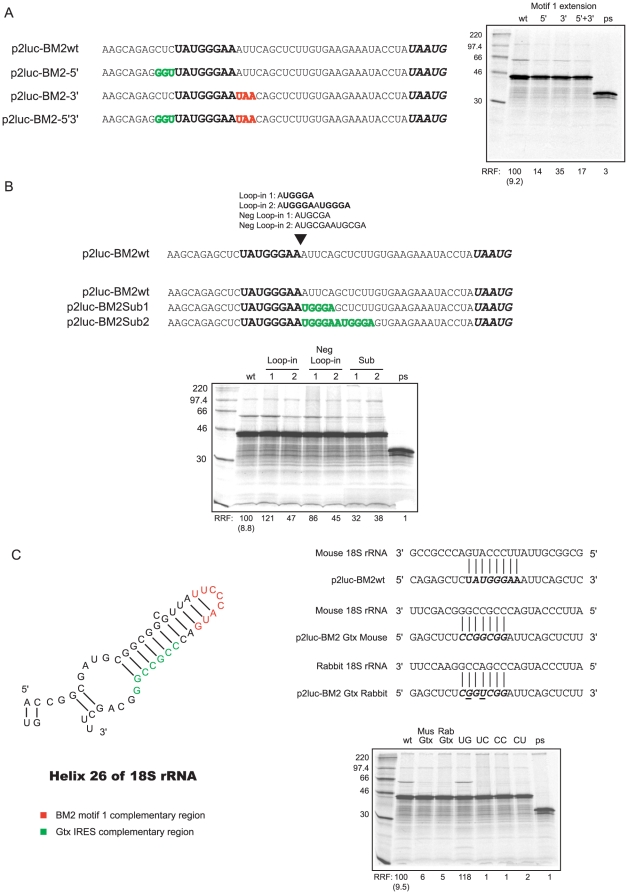
Mutational analysis of the putative interaction between motif 1 and 18S rRNA. (A) Effect of increasing complementarity between motif 1 and 18S rRNA helix 26. The minimal 45 nt TURBS of p2luc-BM2wt is shown with rRNA-complementary bases of motif 1 in bold. The **UAA** UG overlap is in bold italics. Mutations increasing complementarity to 18S rRNA are in outline font, placed 5′ (in green), 3′ (in red) or both 5 and 3′ (in green and red respectively) with respect to motif 1. RNAs (including a control mRNA [ps]) were translated and analysed as in the legend to Figure 1. (B) Effect of increasing motif 1 copy number. Sequences shown above the p2luc-BM2wt sequence were inserted at the position indicated by the large arrow; those below show introduced nucleotide substitutions (in green and bold) in bases adjacent to motif 1. RNAs were translated and analysed as in the legend to Figure 1. (C) Effect of replacing motif 1 with the Gtx IRES-element. Left panel: The secondary structure of the murine helix 26 of the 18S rRNA is shown. The proposed motif 1 binding site is highlighted in red, the Gtx binding region in green. Upper right panel: The p2luc-BM2wt and p2luc-BM2Gtx sequences in the vicinity of motif 1 are shown, and their base pairing to 18S rRNA indicated. In the p2luc-BM2Gtx mutants, the 7 nt core of motif 1 (bold italics in p2luc-BM2wt) was substituted for nucleotides complementary to the region of 18S rRNA where the Gtx IRES is believed to bind. Note the p2luc-BM2Gtx Rabbit sequence is altered from that of the p2luc-BM2Gtx Mouse reporter such that 100% complementarity with the different rabbit 18S rRNA could be achieved. Nucleotides differing between Gtx Mouse and Gtx Rabbit are underlined. RNAs were translated and analysed as in the legend to Figure 1. A series of previously described motif 1 mutants [11] were also translated as controls.

The dependence of BM2 synthesis on interactions between the mRNA and 18S rRNA raises the possibility that increasing 18S rRNA complementarity within motif 1 would lead to increased synthesis of the BM2fluc polypeptide. To test this, the bases adjacent to motif 1 were substituted such that the complementarity to 18S rRNA was increased to the 5′, to the 3′ or in both directions (Figure 3A). In all cases, however, the substitution mutations reduced the frequency of termination-reinitiation events, albeit to a lesser extent with the 3′ complementary extension mutant (Figure 3A).

We went on to examine the possibility that multiple copies of motif 1 would stimulate termination-reinitiation, through the provision of an increased number of tethering sites. As a precedent for this, the murine Gtx mRNA (also known as Nkx 6-2) contains a short 9 nt IRES element that is known to act more efficiently when present in multiple copies. Similarly to motif 1, ribosomes are recruited through interactions between the Gtx IRES and helix 26 of the 18S rRNA [28]–[30], although with the arm of the helix [31] rather than the apical loop (Figure 3C, left panel). Two sets of constructs were prepared. In the first, one or two additional copies of the 6 nt (AUGGGA) core of motif 1 were introduced alongside the original (Figure 3B). Control constructs were also generated in which one or two copies of the hexanucleotide AUGCGA were looped in as a negative control, since this motif 1 mutation was shown previously to be unable to support termination-reinitiation, presumably due to the abolition of mRNA:rRNA base pairing [11]. In the second set of constructs, the bases downstream of the wild-type AUGGGA sequence were substituted to generate one or two extra copies of the AUGGGA motif (Figure 3B). *In vitro* translations revealed, however, that none of the mutations had a stimulatory effect on reinitiation on the BM2 ORF. Looping in a single extra copy of the AUGGGA (or AUGCGA) hexanucleotide had very little effect on the process, whilst insertion of two copies led to inhibition in both contexts (Figure 3B). In constructs where motif 1 was duplicated by virtue of nucleotide substitutions, termination-reinitiation was inhibited to a similar extent in constructs containing either one or two copies of the 18S complementary motif (Figure 3B). These data indicate that there is no additive effect on termination-reinitiation of adding extra copies of the 18S complementary region. In fact, the inhibitory effects seen most likely reflect the importance of the precise spacing of the ribosome with respect to motif 1 (in the loop-in mutants) or, alternatively, some effect on TURBS RNA secondary structure.

As discussed above, the Gtx mRNA is thought to be able to recruit ribosomes by virtue of interactions between helix 26 of the 18S rRNA and the mRNA [28]–[30]). It was therefore of interest to determine whether the minimal 7 nt Gtx motif could functionally replace motif 1. Two constructs were generated, one in which the inserted Gtx element was complementary to murine 18S rRNA (which would result in a mismatch with helix 26 of rabbit 18S rRNA) and one with full complementarity to the rabbit 18S rRNA, such that efficient tethering would be expected in rabbit reticulocyte lysates (RRL; Figure 3C, top-right panel). Termination-reinitiation was found to be inhibited in either mutant and to a similar extent as mutants in which a single nucleotide substitution was present to abolish 18S rRNA:mRNA base pairing (Figure 3C; the single nucleotide mutants have been described previously [11]). These results suggest that motif 1 and the Gtx IRES element cannot functionally replace each other.

### Termination-reinitiation in yeast cells: further evidence for mRNA:18S rRNA interactions in ribosome tethering

**Figure 4:**
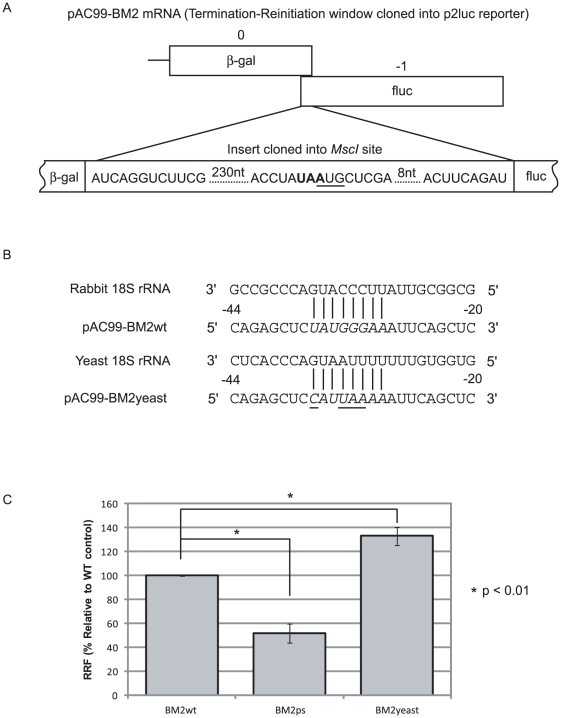
18S rRNA-mRNA base pairing stimulates termination-reinitiation in yeast cells. (A) Schematic showing the yeast dicistronic reporter pAC99-BM2. Cap-dependent translation results in synthesis of β-galactosidase (β-gal), termination-reinitiation gives rise to the synthesis of firefly luciferase (fluc). (B) Sequences of mammalian and yeast 18S rRNAs and their complementarity to the BM2-wt and BM2-yeast reporter mRNAs. The bases mutated to make motif 1 complementary to the yeast 18S rRNA are underlined. (C) Relative recoding efficiencies (relative to BM2-wt, expressed as mean ± SE) as assessed in relation to an in-frame control plasmid. p values were determined by one-tailed *t*-test and are indicated on the figure.

It is known that nucleotide substitutions within motif 1 that are predicted to destabilise the motif 1∶18S rRNA interaction are inhibitory to termination-reinitiation, but it is possible that these mutations may affect TURBS structure or interaction with other translational components, like eIF3 [17]. In an attempt to resolve this issue, we assayed termination-reinitiation in yeast cells, whose 18S rRNA helix 26 equivalent has a somewhat different primary sequence. This experiment was carried out in the context of the yeast dual reporter vector pAC99, into which we had cloned the relevant BM2 information appropriately framed between β-galactosidase and firefly luciferase reporter genes. In pAC99-BM2wt, β-galactosidase is synthesised by cap-dependent translation, whereas firefly luciferase should only be synthesised following reinitiation on the BM2fluc ORF (Figure 4A). To confirm that firefly luciferase was being synthesised as a result of a genuine termination-reinitiation event (as opposed to internal ribosome entry, for example), we generated a premature stop (ps) mutant (pAC99-BM2-ps) in which a stop codon was inserted into the M1 sequence 233 nt upstream of the start codon of BM2fluc. In pAC99-BM2-yeast, motif 1 was changed to give full complementarity to the yeast 18S rRNA partner (Figure 4B). The plasmids were transformed into yeast strain Y349, and β-galactosidase and luciferase assays were carried out. Termination-reinitiation frequency was determined in comparison to the reporter gene activities observed in a vector in which the reporter ORFs were fused in-frame (pAC99-BM2IFC). As expected, termination reinitiation was significantly (p<0.01) reduced in pAC99-yeast-ps, although there was still some background synthesis of fluc (Figure 4C). Importantly, mutation of the motif 1 homologue such that it was fully complementary to yeast 18S rRNA led to a significant (p<0.01) increase in BM2fluc synthesis relative to the wild-type BM2 reporter (Figure 4C), supporting the view that mRNA:rRNA base pairing is a key determinant in BM2 ORF expression.

### Effect of M1 stop and BM2 start codon proximity on termination-reinitiation

**Figure 5:**
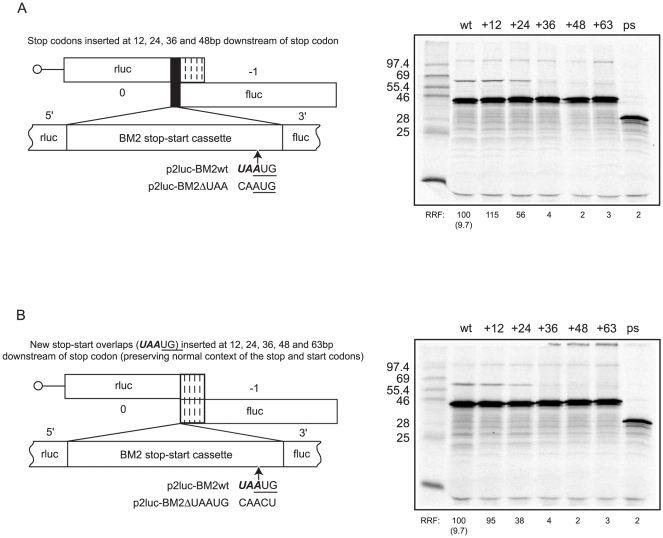
Effect on termination-reinitiation of altering the position of the reinitiation overlap window. (A) The rlucM1/BM2fluc overlap window was changed from **UAA** UG to CAAUG such that termination occurs at the next in-frame stop codon, some 63 nt downstream of the original stop site (+63). New stop codons were then inserted, preserving the stop codon context, at +12, +24, +36 and +48 nt downstream of the natural termination site. Messenger RNAs derived from these plasmids and a control mRNA (ps) were translated and analysed as detailed in the legend to Figure 1. This experiment is a repeat of that found in [11] and is shown here as a control. (B) The rlucM1/BM2fluc overlap window was changed from **UAA** UG to CAACU, and the **UAA** UG sequence reintroduced at +12, +24, +36, +48 or +63 nt downstream preserving both the initiation codon and stop codon context. Messenger RNAs derived from these plasmids were translated and analysed as above.

It has been suggested previously that termination-reinitiation is dependent on the stop codon of the uORF and the start codon of the downstream ORF lying in close proximity [8], [11], [15], [17]–[19], [32], [33]. Indeed, movement of the M1 stop codon more than 24 nt downstream of its original context in the termination-reinitiation ‘window’ of the BM2 signal results in inhibition of reinitiation on the BM2 ORF [11]. However, in all previous investigations into proximity effects of the start and stop codons, the termination site was moved downstream of the start codon of the upstream ORF [8], [11], [15], [17]–[19], [32]). Given that termination-reinitiation almost certainly depends on interactions between the terminating ribosome and the TURBS, it is conceivable that these experiments have overestimated the importance of the relative proximity of the stop and start codons and that the distance between the terminating ribosome and the TURBS is the crucial issue. To investigate this, a series of mRNAs were prepared in which the **UAA** UG overlap was moved downstream *en masse* such that the distance between the stop and start codons was conserved but the ribosome would terminate at varying distances relative to the TURBS (Figure 5B). A series of control mRNAs were also examined in which the stop codon alone was shifted downstream of the BM2 start codon (Figure 5A; details in [11]). In all constructs, nucleotides at –3 and +4 relative to the BM2fluc AUG were maintained to control for context effects of the start codon. As observed previously in the control series [11], reinitiation occurred efficiently when the stop codon was moved up to 24 nt downstream of its original placement but was abolished when the stop was moved further (Figure 5A). Importantly, BM2 translation followed the same pattern in mRNAs where the **UAA** UG motif was moved as a unit, with ablation of synthesis observed when this sequence was moved any further than 24 nt downstream of its original site (Figure 5B). These experiments reveal that the placement of the 40S subunit relative to the TURBS, post-termination, is quite critical.

### Effect of BM2 start codon movement on termination-reinitiation

**Figure 6:**
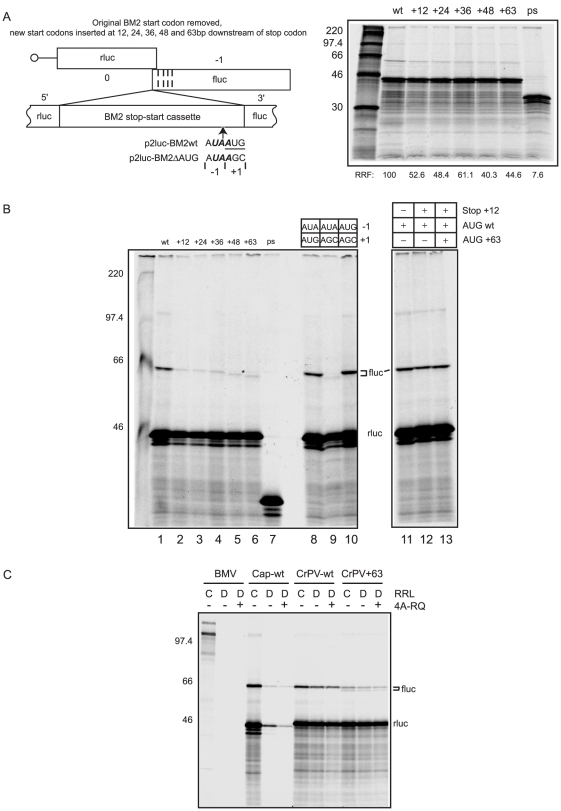
Effect on termination-reinitiation of altering the BM2 start codon position. (A) The termination-reinitiation overlap was mutated from A**UAA** UG to A**UAA**GC and the BM2fluc start codon reintroduced at +12,+24, +36, +48 or +63 nt, preserving the start codon context at the -3 and +4 positions. The codon immediately 5′ of the native reinitiation site is denoted -1, the reinitiation site is shown as +1. Messenger RNAs derived from these plasmids were translated and analysed as above. (B) Left panel: Translations were performed as in Figure 6A and the samples were separated by SDS-PAGE and autoradiographed. The putative AUA reinitiation product is marked with an asterisk. In addition, the three right hand lanes of the left panel show translations of mutant mRNAs in which the codon -1 of the AUG start codon and the start codon (+1) were mutated to the indicated codons in the context of the BM2start +63 RNA. Lane numbers are shown below the figure. Right panel: Translations of RNAs containing stop and start codons at the indicated positions. C) Capped BMV (BMV) RNAs, BM2 wt reporters (Cap-wt), CrPV-IRES driven BM2wt (CrPV-wt) and BM2start +63 (CrPV+63) RNAs were translated in control (C) and eIF4G-depleted (D) RRLs in the absence or presence of ∼1 µM dominant-negative eIFA (4A-R362Q). RNAs were translated as described in [26] and analysed as above.

As reinitiation is not absolutely dependent on the close proximity of stop and start codons (Figure 5A), it seemed conceivable that termination-reinitiation could still occur efficiently if the start codon of the BM2fluc ORF was moved downstream of the stop codon of rlucM1. A series of mRNAs were prepared in which the original BM2fluc start codon was mutated to AGC and new AUG restart codons inserted independently at +12, +24, +36, +48 and +63 nt, again preserving the wild-type Kozak consensus. As can be seen in Figure 6A, movement of the start codon to +12 resulted in 50% inhibition of fluc synthesis, but there was no further reduction in product yield when the restart codon was displaced further downstream. However, the electrophoretic mobility of this reinitiation product did not show the clear increase that would be expected given that the restart product would become progressively smaller with increased displacement of the restart codon. Rather, the reinitiation product band seemed less sharp and more diffuse with the +48 and +63 mRNAs. This suggested the possibility that reinitiation might be occurring at two sites: one in the region of the wild-type reinitiation site (and so necessarily at a non-AUG codon), and the other at the displaced AUG restart codon.

The most obvious potential non-AUG codon for the first type of reinitiation event is the AUA codon located immediately upstream (-1 position) of the wild-type reinitiation site at +1 (Figure 6A). We therefore tested whether reinitiation can indeed occur at this -1 position, by generating an AUG in this position and removing the wild-type +1 AUG. The results showed that the efficiency of reinitiation at an AUG in the -1 position was similar to when it was in the normal (+1) position (Figure 6B, compare lanes 8 and 10), in agreement with the finding that reinitiation efficiency in the RHDV system was only slightly compromised if the restart codon was moved one codon upstream [19]. Given that quite efficient reinitiation still occurs in FCV and BM2 RNAs when the wild-type AUG is mutated to non-AUG codons [11], [15], [17], this observation suggests that when the wild-type +1 AUG has been mutated, there is a strong possibility of some reinitiation occurring at the -1 AUA codon.

As for reinitiation at the displaced AUG codon, the data of Figure 6B (lanes 1–6) demonstrate that reinitiation certainly occurs at these downstream sites, because on this higher resolution gel the product size clearly decreases as the displacement is increased, although the efficiency is only 20–25% of the reinitiation frequency in the wild-type mRNA (lane 1). In fact, in this particular experiment there is much more downstream cistron product from the displaced AUG than product from the putative -1 AUA reinitiation site, which was only just visible on the autoradiogram (highlighted by the asterisk). In reviewing the results of many experiments of this type we conclude that the relative use of the two potential reinitiation sites shows quite a large degree of variability, which seems related to the use of different batches of reticulocyte lysate and so may possibly be due to small batch to batch differences in the endogenous ionic conditions. The variability seems to mainly affect the yield of putative -1 AUA reinitiation product, whereas the yield of product from the displaced AUG codon is relatively invariant at ∼20% of the wild-type mRNA yield. The two extremes of the variability range are well illustrated by Figure 6B (very minimal use of the -1 AUA site) and Figure 6C, where there is actually more putative reinitiation at the -1 AUA than at the displaced AUG codon.

The fact that reinitiation, albeit at reduced efficiency, can occur when the first AUG is moved 63 nt downstream raises the question of whether the reinitiating 40S subunits access this site by linear scanning from the TURBS. We examined this by exploiting the dependence of ribosomal scanning on eIF4G [34]. The BM2wt and BM2start +63 mRNAs were modified such that translation of the upstream M1 ORF was driven by the cricket paralysis virus (CrPV) IRES, which does not require eIF4G for function. These mRNAs (CrPV-BM2wt, CrPV-BM2start+63) were then translated alongside capped brome mosaic virus RNAs (BMV, Promega) and BM2wt RNAs (as a control for the efficacy of eIF4G depletion) in mock- and eIF4G-depleted rabbit reticulocyte lysates (Figure 6C). Translations were also performed in the absence or presence of dominant-negative eIF4A (eIF4A R362Q [35]) to inhibit the activity of any residual eIF4G that had escaped depletion. As expected, the translation of the capped wild-type RNA (BM2wt) and BMV RNAs were inhibited in the eIF4G-depleted RRL, but the CrPV IRES-containing mRNAs were translated efficiently in both control and depleted RRL (Figure 6C). Importantly, depletion of eIF4G had no effect on the reinitiation efficiency in either the CrPV-BM2wt or CrPV-BM2start +63 mRNAs, suggesting that the reinitiation site can be located in a scanning-independent manner.

When two AUG codons were present, one at +63 and the other at either the +1 (wild-type) or -1 position, the upstream site took complete precedence over the +63 site (Figure 6B, lanes 8 and 10), and this precedence was maintained even when the stop codon was moved 12 codons downstream (Figure 6B, lanes 11–13). Thus the strongly preferred site for reinitiation is an AUG codon just downstream of the TURBS. If there is no AUG in this region, the reinitiation mechanism apparently seeks alternatives: either an acceptable non-AUG codon in this same region or an AUG codon further downstream. Reinitiation in the later case does not involve eIF4G/4A-dependent linear scanning, and so it is more likely to involve a direct transfer of the 40S subunit from the TURBS to the AUG, a transfer which might be facilitated by looping out the mRNA between the tethered 40S ribosomal subunit and the AUG.

### The involvement of eIF3 in termination-reinitiation in influenza BM2 expression

**Figure 7:**
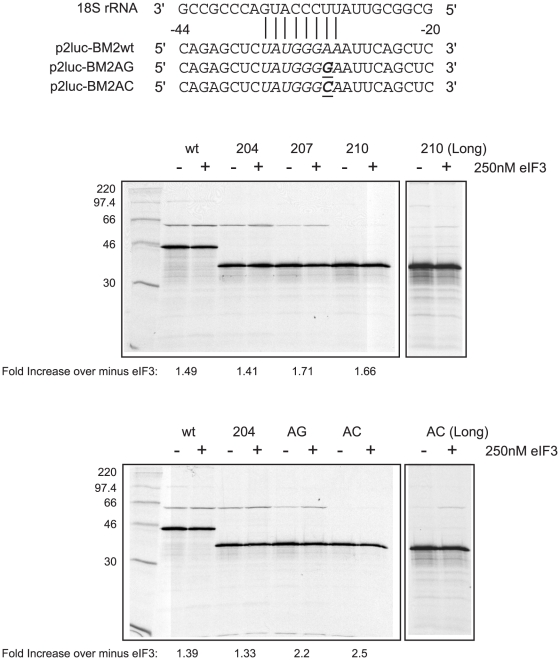
eIF3 stimulates reinitiation on the BM2 ORF. Top: The sequence of two motif 1 mutants and their putative interactions with 18S rRNA are shown. Bottom: A series of TURBS deletion mutants (204, 207, 210; described in [11]) and the two motif 1 point mutants above were translated in Flexi® RRL alongside the positive control BM2-wt mRNA in the absence or presence of 250 nM eIF3. Messenger RNAs derived from these plasmids were translated and analysed as in the legend to Figure 1. Longer exposures of a portion of the autoradiograph are shown on the right.

Studies of the FCV signal have indicated a role for eIF3 in termination-reinitiation as, for example, addition of supplementary eIF3 to RRL is able to specifically stimulate synthesis of the ORF3 reinitiation product [17]. Furthermore, greater stimulation is observed with mRNAs that show a partial defect in reinitiation in the absence of eIF3, suggesting that the increase in eIF3 concentration may rescue the negative phenotype, perhaps by allowing increased binding of eIF3 to the mRNA [17]. To analyse whether the same is true of reinitiation on the BM2 ORF, we examined the effect of exogenous eIF3 on reinitiation on mRNAs containing a fully functional signal (BM2wt, BM2-204), or mutants defective by virtue of a deletion (BM2-207, BM2-210, described in [11]) or a substitution in motif 1 (BM2-AG, BM2-AC). To focus specifically on the effect of eIF3 on termination-reinitiation rather than 5′-dependent initiation, the BM2 sequences were sub-cloned such that the rlucM1 ORF would initiate on an alanine codon provided as part of the CrPV IRES. As can be seen in Figure 7, exogenous eIF3 specifically stimulated synthesis of the BM2fluc reinitiation product with all of the mRNAs tested, including those mutants that were essentially inactive (BM2-210 and BM2-AC; most evident in long exposures, see right panel, Figure 7). Importantly, the addition of an unrelated, similarly purified protein had no effect on reinitiation in any of the RNAs described above (Figure S1), further highlighting the specificity of eIF3′s effect on reinitiation.

### Termination-reinitiation on the BM2 ORF may be dependent on RNA secondary structures in the TURBS

**Figure 8:**
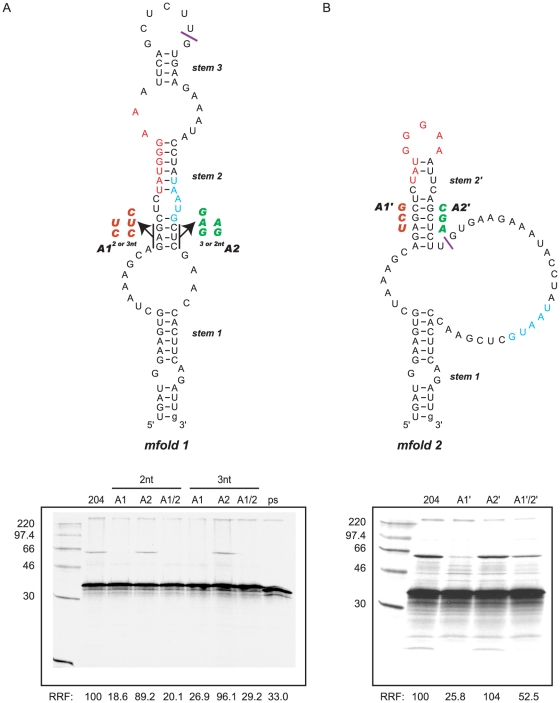
Mutational analysis of putative stem regions that may form in the BM2 TURBS. (A) The mfold 1 structure (from [11]) is shown with motif 1 shown in red, the termination-reinitiation overlap shown in cyan and the sequences likely to be occupied by a ribosome terminating at the M1 ORF are 3′ of the purple line. Mutations were introduced at the bottom of stem 2, with both the 2 nt and 3 nt substitution mutations shown next to arm 1 (A1) or arm 2 (A2). Messenger RNAs derived from these plasmids and control mRNA (204, ps) were translated and analysed as detailed in the legend to Figure 1. RRF denotes the relative reinitiation frequency adjusted for relative methionine content as compared to the wt (set as 100%). (B) The mfold 2 structure (from [11]) is shown with motif 1, the termination-reinitiation overlap, and the ribosome protected region marked as above. Mutations were introduced at the bottom of stem 2′ and are shown next to arm 1 (A1′) or arm 2 (A2′). Messenger RNAs derived from these plasmids and a control mRNA (204) were translated and analysed as above. RRF denotes the relative reinitiation frequency adjusted for relative methionine content as compared to the wt (set as 100%).

Given the likely importance of the interaction between motif 1 and 18S rRNA, we previously carried out RNA structure mapping and minimal free energy predictions to assess whether motif 1 was in base-paired or single stranded conformation in the native mRNA. This work implicated two potential structures, mfold 1 and mfold 2 (Figure 8A; [11]). In mfold 1 the 18S complementary region is sequestered in a base-paired region of stem 2. We previously hypothesised that translation through the segment 7 mRNA and termination of ribosomes at the M1 stop codon would prevent these interactions, creating a structure similar to mfold 2 (Figure 8B). In this structure, the 18S complementary region would then be presented to helix 26 of the 18S rRNA on the apical loop of a metastable stem-loop structure.

Given that translation through the TURBS up to the termination-reinitiation window is a prerequisite for efficient reinitiation [11], one can speculate that mfold 2 is the biologically relevant fold, and that destabilisation of the central stem of mfold 1 (stem 2) would have little effect on the reinitiation process. To test this hypothesis we created destabilising mutations within either arm of stem 2 (chosen to avoid disruption of the 18S rRNA complementary region) in the context of the p2luc-BM2-204 parental plasmid (which encodes the minimal required region for termination-reinitiation [11]). The mutations created were of either 2 or 3 nt to control for context effects on the BM2 AUG, creating the arm 1 mutants p2luc-BM2A1-xnt, and the arm 2 mutants p2luc-BM2A2-xnt (where x =  the number of nucleotides mutated). We also prepared double mutants (A1/2-xnt) to create a pseudowild-type structure where base-pairing between the two arms is restored. In the context of both the 2 nt and 3 nt mutations, substitution of bases in arm 1 abolished termination-reinitiation, however substitution of the bases at the bottom of arm 2 had little effect on BM2fluc synthesis relative to the BM2-204 control. The double pseudowild-type mutation also demonstrated abolition of BM2fluc expression (Figure 8A). Taken together these results suggest that the structure presented in mfold 1 is unlikely to play a role in termination-reinitiation as disruption of arm 2 of stem 2 has little effect on BM2 translation, and no restoration of BM2 synthesis was observed in the pseudowild-type construct.

As opposed to mfold 1, the structure presented for mfold 2 would still be able to form when the ribosome has translated through the upstream ORF and is in the process of termination. We tested this structure in the same way, by introducing destabilising mutations independently in both arms at the base of stem 2′ (creating mutants p2luc-BM2A1' and p2luc-BM2A2′) and preparing a double mutant that would yield a pseudowild-type structure, which would be expected to exhibit BM2fluc synthesis (p2luc-BM2A1′/2′). It should be noted that the A1′ mutation is very similar to that of the A1-2/3 nt mutant, given that this arm base pairs to form both stem 2 and stem 2′ in the two putative folds. As expected, this mutation dramatically inhibited synthesis of BM2fluc similarly to that observed with the A1 mutation (Figure 8B). However, as before, no inhibition of BM2 synthesis was observed when stem 2 was destabilised in the 3′ arm (Figure 8B). Nevertheless, the partial restoration of BM2 expression (to around 50% of wild-type) seen with the A1′/2′ double mutant, indicates that base-pairing in this region may play some role in termination-reinitiation. The simplest conclusion from these experiments, however, is that neither mfold 1 nor mfold 2 represent the sole active confirmation, although it is possible that these mutants may form another structure that can present the TURBS motif 1 to helix 26 of 18S rRNA.

## Discussion

Previous work has revealed that termination-dependent reinitiation on the BM2 ORF of segment 7 of influenza B virus is dependent on a relatively short (45 nt), largely unstructured, TURBS containing a typical motif 1 element towards the 5′ end [11]. In the present study, we investigated features of the TURBS required for efficient reinitiation, focusing primarily on the proposed interaction between TURBS motif 1 and helix 26 of 18S rRNA. Whilst the effects of point mutations within motif 1 are consistent with such an interaction, direct evidence is lacking in the BM2 system. We began by attempting to block the interaction by targeting the binding partners with antisense oligonucleotides. Such an approach was successfully employed in studies of the IRES-like properties of the Gtx leader (which recruits ribosomes *de novo* by virtue of interactions between the 18S rRNA and the mRNA) and revealed that the IRES activity could be blocked by AONs that bind either the mRNA or the rRNA [36]. Whilst we were able to observe specific inhibitory effects with an oligonucleotide that targeted motif 1, no effect was observed with an oligonucleotide targeting the ribosomal RNA (Figure 2). Our failure to observe an effect of the AON complementary to the apical loop of helix 26 may be due to a failure of the AON to access the target. The Gtx mRNA:rRNA interaction occurs at a different region of helix 26 than the putative segment 7 mRNA:rRNA interaction, and this may be more accessible. Indeed, termination-reinitiation could not be reconstituted in reporter mRNAs in which motif 1 was replaced by the Gtx 18S rRNA binding motif (Figure 3C), and thus different responses to oligonucleotides targeting the rRNA may be expected. It may also be that the 18S rRNA-targeting oligonucleotide may need to adopt a similar structure to the mRNA if it is to effectively bind to the 18S rRNA and block reinitiation. The inhibition of termination-reinitiation by the AON that targeted motif 1, in contrast, is highly consistent with its proposed role in binding to helix 26, although effects via RNA conformation or binding to an unknown molecule cannot be ruled out. However, the weight of evidence from mutational analysis, the yeast data described below and the AON titrations strongly suggest that the inhibitory effect of AONs directed against motif 1 are likely due to their blocking of the interaction between mRNA and 18S rRNA.

Subsequently, we sought to confirm the interaction by investigating termination-reinitiation in yeast cells, exploiting the fact that the helix 26 equivalent in yeast 18S rRNA has a primary sequence distinct from that of the rabbit. Using a dicistronic reporter mRNA with variant motif 1 sequences, we found that increasing the complementarity of the motif to that of yeast 18S rRNA stimulated termination-reinitiation, supporting strongly the view that intermolecular interactions are important in reinitiation on the BM2 ORF. However, two aspects of this experiment require comment. First, a high background activity was observed in control assays (Figure 4), the origin of which is uncertain Secondly, in yeast cells, the BM2wt reporter mRNA, whose motif 1 is not fully complementary to the helix 26 target, showed an efficiency of termination-reinitiation only two-fold lower than the BM2yeast mRNA. In RRL, a single base mismatch between mRNA and rRNA is sufficient to inhibit BM2 synthesis 10-20 fold [11]. One possible explanation for this difference is that the higher concentrations of mRNA and rRNA in an intact cell can act to stabilise the (presumably) weakened mRNA:rRNA interaction. During preparation of this manuscript, Luttermann and Meyers (2009) published a similar, more sophisticated, investigation of FCV motif 1:18S rRNA interactions using a yeast expression system in which both mRNA and rRNA partners could be modified. This work provided strong evidence in support of a requirement for mRNA:rRNA interactions in termination-reinitiation in the expression of FCV VP2 [16].

Since the reinitiation mechanism shows a fairly strong preference for an AUG codon, it seems a certainty that initiator Met-tRNA is involved. This, in turn, implies that the ribosome which has just terminated at the stop codon must dissociate into subunits prior to reinitiation, in order to allow eIF2/GTP/Met-tRNA_i_ ternary complex binding to the 40S subunit. So it is a 40S subunit rather than an 80S ribosome that interacts with TURBS motif 1. The fact that reinitiation efficiency decreases quite abruptly with increasing distance of displacement of the stop codon further downstream (Figure 5) favours a model in which this 40S subunit is transferred directly from the termination site to the TURBS, rather than dissociating from the mRNA after termination followed by reassociation with the TURBS.

Recent work has implicated eIF3 in the termination-reinitiation process [17], which is of interest because eIF3 plays an important role in disassembly of ribosomes following the termination event which leaves the 80S ribosome with bound deacylated tRNA and still associated with the mRNA [37], [38]. At low (sub-optimal) Mg^2+^, this disassembly requires eIF1 and eIF3, but no other protein factor. First, eIF3 binds to the solvent face of the 40S subunit, promoting dissociation of the 60S subunit, and leaving the 40S subunit (with bound deacylated tRNA) still associated with the mRNA. Then eIF1 ejects the deacylated tRNA, while the eIF3j subunit promotes dissociation of the 40S/eIF1/eIF3 complex from the mRNA [37]. At higher, more physiologically relevant Mg^2+^, as would pertain in our translation assays, there is also a requirement for ABCE1 and ATP [38], which catalyses the first step of dissociation of the ribosomal subunits, ejecting the 60S subunit and again leaving the 40S subunit (plus bound deacylated tRNA) still associated with the mRNA. Then eIF3 binds to the solvent face of the 40S subunit, thereby preventing any ribosomal subunit reassociation, and events thereafter are exactly as at low Mg^2+^ [37], [38].

Initiation factor eIF3 has been shown to crosslink to the FCV TURBS [17]. More important, supplementary eIF3 has been shown to stimulate reinitiation at the wild-type FCV TURBS, but more especially at TURBS derivatives which are partially defective due to deletions or point mutations [17], just as has been observed here (Figure 7). These results, particularly the stimulation of the partially defective TURBS, have suggested a model in which it is specifically the eIF3 associated with the TURBS that binds those 40S subunits which are destined to reinitiate translation [17]. There is no conflict between this hypothesis and the model in which tethering the 40S subunit to the TURBS is due to base-pairing between TURBS motif 1 and 18S rRNA. eIF3 binds predominantly to the back or solvent face of the 40S ribosomal subunit, and is known to make direct contacts with the 18S rRNA component of the small ribosomal subunit [39], [40]. A bridging interaction in which eIF3 binds simultaneously to the 40S subunit and to the TURBS could help stabilise the binding of the 40S subunit to the TURBS for sufficient time as is required to acquire an eIF2/GTP/Met-tRNA_i_ ternary complex, and other necessary initiation factors. After all, the analogous Shine-Dalgarno interaction of prokaryotic 30S subunits is generally considered to be very transitory unless it is accompanied and stabilised by a P-site fMet-tRNA base-pairing with an AUG (or GUG) at an appropriate distance further downstream.

An important question that remains to be resolved is whether there is a role for TURBS RNA secondary structure. We previously proposed that the TURBS may fold into two main configurations, in the first of which (mfold 1) motif 1 is sequestered in a base-paired region [11], which may explain why it does not have significant IRES activity (as is seen with Gtx [28]–[30]). Translation through and termination at the M1 ORF would remodel mfold 1 to a structure similar to that shown in mfold 2 (Figure 8B) such that motif 1 would then be presented to the 18S rRNA on the apical loop of a stem-loop structure. The experiments described in Figure 1 agree well with dependence on a structure similar to that presented in mfold 2, in that the substituted nucleotides in the 6.1 and 6.4 replacement mutants would act to disrupt the stem, whereas substitution of nucleotides in the 6.6 replacement mutants would be expected to have no effect as they would lie within the mRNA channel of the terminating ribosome. However, we also carried out a mutational analysis of the main stem regions present in mfold 1 and mfold 2 but the data did not corroborate our structural predictions. Whilst disruption of either of the putative stems in one arm inhibited reinitiation, little effect was observed when stem formation was disrupted in arm 2 of either stem-loop structure, although some restoration of BM2 synthesis was observed in an mRNA containing a pseudowild-type mfold 2 structure. It should be noted that RNA structure mapping of the TURBS of BM2 [11], FCV (Brierley *et al.* unpublished observations) and MNV [20] TURBS reveals that they are largely single-stranded, and probably metastable. It may be that TURBS are able to adopt a variety of conformations, some of which are able to facilitate termination-reinitiation, for example the 6 nt replacement mutant 6.4r2 (Figure 1B). Alternatively, the requirement for translation through the TURBS may not be due to a dependence on translational remodelling and ‘unzipping’ of motif 1 from paired regions, but rather just to place the ribosome in proximity to motif 1 (similar to the case for reinitiation in bacteriophage where the ribosome is placed close to the vestigial Shine-Dalgarno [SD] motif [41]). It is clear that further work is required to understand the putative role of RNA secondary structure in termination-reinitiation.

Previous studies on the termination-reinitiation process have suggested the importance of the close proximity of the stop codon of the upstream ORF and the start codon of the downstream ORF [8], [11], [15], [17], [18], [32]. This is believed to reflect a restricted mobility of the tethered ribosome; that is, it may be able to undergo only limited movement following termination. However, we show here that the distance between the terminating ribosome and the TURBS is more critical to reinitiation efficiency (Figure 5) and that when the start codon of the BM2 ORF is placed downstream of the stop codon, reinitiation is detectable even when the start codon is moved up to 63nt downstream of its original position (Figure 6). This suggests that it is not solely the distance between the start and stop codons *per se* that affects reinitiation efficiency but rather how well the ribosome can be tethered (by virtue of the distance between where the ribosome terminates and the TURBS). However, whilst efficient reinitiation requires an AUG at, or very near, the wild-type site (Figure 6A and 6B), other reinitiation sites can be used when the native reinitiation codon is absent, albeit at lower efficiency. Under the latter circumstance, ribosomes can reinitiate at a downstream AUG codon (Figure 6B), or at a near-cognate initiation codon (such as the -1 AUA codon, Figure 6) close to the native reinitiation site. However, if given a choice of a wild-type AUG and one located downstream, the ribosome always selects the wild-type AUG, even if the ribosome artificially terminates downstream of the reinitiation window (Figure 6B), suggesting that the TURBS may cause the tethered 40S subunit to ‘snap back’ to the proper site of reinitiation. Importantly, we show that there is no requirement for eIF4G in location of either wild-type or downstream reinitiation sites. As such, location of downstream AUGs is likely due to the 40S subunit being transferred directly to the AUG whilst tethered to the TURBS (perhaps in complex with eIF3 and other factors) by an RNA-looping mechanism.

## Supporting Information

Figure S1 **Recombinant 4EBP1 has no significant effect on reinitiation on the BM2 ORF.** Motif 1 and TURBS deletion mutants were translated in the absence or presence of 250nM 4EBP1 (details as in Figure 7). The fold-stimulation over the reinitiation levels observed in the absence of 4EBP1 are shown below the autoradiographs.
(EPS)
